# Adaptive enrichment in biomarker-stratified clinical trial design

**DOI:** 10.1186/1745-6215-14-S1-O11

**Published:** 2013-11-29

**Authors:** Deepak Parashar, Jack Bowden, Colin Starr, Lorenz Wernisch, Adrian Mander

**Affiliations:** 1 Cambridge Cancer Trials Centre, Cambridge, UK; 2 MRC Biostatistics Unit, Cambridge, UK

## 

In Phase II oncology trials, targeted therapies are being constantly evaluated for their efficacy in specific populations of interest. Such trials require designs that allow for stratification based on the participants' biomarker signature. One of the disadvantages of a targeted design (defined as enrichment in biomarker-positive sub-population) is that if the drug has at least some activity in the biomarker-negative subjects, then their effect in the biomarker-negative population may never be known.

Jones and Holmgren (JH) have proposed a design to determine whether drug has activity only in target population or the general population. Their design is an enrichment adaptation based on two parallel Simon two-stage designs. Unfortunately, there are several pitfalls in the JH design: the issue of hypothesis testing is not properly addressed and the type I error, power calculations and expected sample size formulae are wrong too.

We study the JH design in detail, appropriately control the type I and type II error probabilities that yield novel optimal designs. We also discuss various alternative Family Wise Error Rates (FWER) and the Individual Hypothesis (IH) error rates in the weak sense as well as the strong sense.

For each option of the error controls, we search for designs over a 10 trillion search space and obtain optimal designs that minimise the expected sample size. For the particular example trial that JH consider, our optimal design requires 38% fewer subjects in comparison with the two parallel Simon two-stage design thereby offering substantial efficiency in terms of the expected sample size. In conclusion, our rectified design provides a robust framework for adaptive enrichment in biomarker-stratified Phase II trial design.

